# Associations between healthy food groups and platelet-activating factor, lipoprotein-associated phospholipase A_2_ and C-reactive protein: a cross-sectional study

**DOI:** 10.1007/s00394-023-03277-8

**Published:** 2023-12-08

**Authors:** Carolyn J. English, Mark Jones, Anna E. Lohning, Hannah L. Mayr, Helen MacLaughlin, Dianne P. Reidlinger

**Affiliations:** 1 Faculty of Health Sciences and Medicine, Bond University, Robina, QLD Australia; 2 Faculty of Health Sciences and Medicine, Institute of Evidence-Based Healthcare, Bond University, Robina, QLD Australia; 3 Department of Nutrition and Dietetics, Princess Alexandra Hospital, Woolloongabba, QLD Australia; 4 Centre for Functioning and Health Research, Metro South Hospital and Health Service, Brisbane, QLD Australia; 5 Faculty of Health, School of Exercise and Nutrition Sciences, Queensland University of Technology, Brisbane, Australia; 6 Nutrition Research Collaborative, Royal Brisbane and Women’s Hospital, Brisbane, Australia

**Keywords:** Inflammation, Cardiovascular diseases, Diet, Healthy, Dietary plant protein, Biomarker, COVID-19

## Abstract

**Purpose:**

To investigate the association between pro-inflammatory markers platelet-activating factor (PAF), lipoprotein-associated phospholipase A_2_ (Lp-PLA_2_), hsCRP, and intake of core food groups including fruit, cruciferous and other vegetables, grains, meat and poultry, fish and seafood, nuts and legumes, and dairy.

**Methods:**

A cross-sectional study was conducted. 100 adults (49 ± 13 years, 31% male) with variable cardiovascular disease risk were recruited. Data were collected in 2021 and 2022. Fasting PAF, Lp-PLA_2_ activity, hsCRP and usual dietary intake (via a validated food frequency questionnaire) were measured. Intake of foods were converted into serves and classified into food groups. Correlations and multiple regressions were performed with adjustment for confounders.

**Results:**

A one-serve increase in cruciferous vegetables per day was associated with 20–24% lower PAF levels. An increase of one serve per day of nuts and legumes was associated with 40% lower hsCRP levels. There were small correlations with PAF and Lp-PLA_2_ and cheese, however, these were not significant at the Bonferroni-adjusted *P* < 0.005 level.

**Conclusion:**

The lack of associations between PAF and Lp-PLA_2_ and other healthy foods may be due to confounding by COVID-19 infection and vaccination programs which prevents any firm conclusion on the relationship between PAF, Lp-PLA_2_ and food groups. Future research should aim to examine the relationship with these novel markers and healthy food groups in a non-pandemic setting.

**Supplementary Information:**

The online version contains supplementary material available at 10.1007/s00394-023-03277-8.

## Introduction

Diet is a modifiable risk factor associated with cardiovascular disease (CVD) and an optimal intake of healthy foods including whole grains, vegetables, fruits, nuts, legumes, dairy, and fish has been shown to reduce CVD risk by as much as 65% [1]. This reduction in risk may be partly due to the anti-inflammatory potential of these foods as atherosclerosis, the underlying cause of CVD, is a chronic inflammatory disease of the arteries, and healthy food groups have been found to modulate this inflammation [2–4]. Chronic inflammation is traditionally measured by high sensitivity C-reactive protein (hsCRP), however, the use of this biomarker is not without limitations. CRP is a nonspecific marker of inflammation and can be elevated in acute inflammatory conditions [5]. There is a high intra-person variability with CRP requiring repeat measurements to gain an accurate assessment of true levels [6]. In addition, new research has discovered that CRP has several isoforms, some atherogenic and some protective and current assays are not able to differentiate between the two [7]. Thus, researchers have been looking for other biomarkers, especially those specific to endothelial dysfunction, to detect and monitor chronic inflammatory processes. Two novel markers involved in CVD that are receiving increasing attention due to their association with endothelial inflammation are platelet-activating factor (PAF) and lipoprotein-associated phospholipase A_2_ (Lp-PLA_2_) [8].

PAF is an ether-linked glycerophospholipid that is one of the most potent inflammatory mediators in the body that is active in nanomolar concentrations and is closely implicated in all stages of atherosclerosis [9]. PAF is produced by numerous cells such as platelets, endothelial cells, and leukocytes and triggers an inflammatory cascade through the act of binding to the G-protein coupled PAF receptor [10, 11]. PAF is involved in the early stages of atherosclerosis by the mediation of adhesion of monocytes to the endothelium and increasing endothelial permeability, allowing low-density lipoproteins (LDL) and monocytes to migrate into the intima [12, 13]. PAF is also responsible for stimulating reactive oxygen and nitrogen species which contributes to the oxidation of LDL once inside the intima [14]. PAF stimulates the release of numerous cytokines such as interleukin-6 (IL-6), interleukin-8 (IL-8), tumour necrosis factor alpha (TNF-α) and monocyte chemotactic protein (MCP-1) [12, 15, 16]. PAF further stimulates the differentiation of monocytes into macrophages which engulf the oxidised LDL to create foam cells [17, 18]. It is also involved in later stages of atherosclerosis through the stimulation of plaque growth and their eventual rupture or thrombosis [19]. PAF has been shown to be associated with many CVDs including coronary heart disease, acute myocardial infarction, heart failure and stroke [20–23]. PAF is further involved in other metabolic chronic diseases such as diabetes and non-alcoholic fatty liver disease [24–26].

Lp-PLA_2_ is a 50-kD, Ca^2+^ independent phospholipase (EC 3.1. 1.47) that is classified within Group VIIA of the PLA_2_ superfamily [27, 28]. Lp-PLA_2_ catalyses the hydrolysis of the *sn*-2 ester bond of glycerophospholipids such as the acetyl group on PAF and is actively involved in PAF metabolism [29]. However, Lp-PLA_2_ is not specific to PAF, and because of the capacity of its active site, can also accommodate oxidatively truncated fatty acids at the sn-2 position, thus hydrolyses oxidised phospholipids on the surface of LDL particles [30]. This hydrolysis reaction results in the generation of two atherogenic by-products lysophosphatidylcholine (LysoPC) and oxidized, nonesterified fatty acids (OxNEFA) [31]. These by-products contribute to endothelial dysfunction, inflammation and plaque instability by upregulating adhesion molecules, acting as a chemoattractant to monocytes, activating leukocytes, and stimulating cytokine production such as IL-6 and TNFα [31–34]. Lyso PC also upregulates osteogenic genes and increases calcification in vascular smooth muscles cells [35] and is responsible for inducing smooth muscle migration into the intima [36]. Lp-PLA_2_ has a low biological fluctuation unlike CRP, and is a vascular specific marker, with higher levels correlated with plaque instability [37]. It has been shown to be associated with numerous CVDs in a manner similar to PAF, including coronary heart disease, stroke, and calcific aortic valve stenosis as well as type 2 diabetes and chronic kidney disease [38–43].

The Mediterranean Diet and its individual components have been widely researched in relation to PAF and Lp-PLA_2_ [44]. Less investigated are a priori non-Mediterranean dietary patterns with these biomarkers, however, two recent reviews have reported on Mediterranean and other healthy dietary patterns and PAF and Lp-PLA_2_ including a dietary pattern consistent with national dietary recommendations [8, 45]. Whilst previous research has explored foods and food groups and their association with inflammation and in particular CRP, studies looking at food groups and PAF and Lp-PLA_2_ are limited. Some reviews have focused on individual foods or nutrients and their association with PAF and Lp-PLA_2_ [11, 44]; however, many of the studies included in these reviews were in vitro or in animal models*.* Other studies that have examined PAF and/or Lp-PLA_2_ in humans did not utilise strict exclusion criteria to prevent confounding [20, 46–50] with the exception of one study specifically investigating the Mediterranean Diet and PAF and its enzymes in healthy adults [51]. For example, numerous medications and supplements have been shown to lower levels of PAF and Lp-PLA_2_ such as statins, ezetimibe, fenofibrate, niacin, orlistat, hormone replacement therapy, omega-3 fatty acids, and fish oils; and smoking has been shown to raise levels [52–56]. The inclusion of participants who smoke or who are taking these medications and/or supplements may introduce confounding into a study preventing a true understanding of the relationships between foods and the markers of inflammation. In addition, several ethnic groups such as Asians and Africans have been shown to have lower levels of Lp-PLA_2_ due to genetic polymorphisms [57–59].

Furthermore, many studies of Lp-PLA_2_ have measured plasma concentration (mass) instead of enzyme activity. Enzyme activity assays have now replaced mass assays as they are a more robust measurement of Lp-PLA_2_ and provide better risk stratification. Lp-PLA_2_, once secreted by the macrophages, is carried bound both to high-density lipoprotein (HDL) and LDL with the Lp-PLA_2_ bound to HDL thought to be protective [31]. Mass assays only detect a small portion of the total Lp-PLA_2_, predominantly the Lp-PLA_2_ associated with HDL [60]. Enzymatic assays that measure Lp-PLA_2_ activity capture the Lp-PLA_2_ bound to LDL cholesterol which is more atherogenic [61].

Therefore, this study aimed to examine the association of core food groups, aligned with the Australian dietary guidelines [62], with PAF, Lp-PLA_2_ activity and hsCRP in a broadly Caucasian population at varying risk of CVD, utilising strict exclusion criteria.

## Materials and methods

Methodology for this study, except for the assessment of dietary intake and calculation of servings of food groups, has been previously published [63].

### Study design and setting

This cross-sectional study was carried out on the Gold Coast, Queensland Australia and used a convenience sampling technique. Participants were recruited through community-based organisations such as fitness centres, surf lifesaving clubs, sporting clubs, council libraries, community centres, shopping centres, a university setting, and through social media and online/email methods to obtain a representative community sample of healthy adults at varying risk of CVD. The study began recruitment in February 2021 and samples were collected from May 2021 to April 2022, over four 2-week periods.

Approval for this study protocol was obtained from the Bond University Human Research Ethics Committee (approval number DR03194) and the study conforms to the ethical guidelines of the 1964 Declaration of Helsinki and its later amendments. All participants provided written informed consent before taking part in the study.

### Study population and sample size

Eligible participants included adults who were classified as either high or low risk of CVD and were aged 18–70 years old. Participants had to either have confirmed type 2 diabetes OR have two or more of the following risk factors for CVD: systolic blood pressure ≥ 140 mm Hg or diastolic ≥ 90 mm Hg or receiving medication for high blood pressure; total cholesterol ≥ 5.2 mmol/L; LDL cholesterol ≥ 4.1 mmol/L; HDL cholesterol ≤ 1 mmol/L; family history of premature coronary heart disease (CHD) (≤ 60 years); or excess weight, BMI ≥ 25 kg/m^2^ in order to be classified as high risk of CVD. Participants had to report the absence of any chronic disease, not be on any routine medication, be below the cut-offs listed for high-risk individuals for BMI, blood pressure, cholesterol and report no family history of premature CHD in order to be classified as low risk of CVD.

Any participant who reported a history of angina, peripheral vascular disease, myocardial infarction, congenital heart disease, or stroke, or were current smokers were excluded. Participants who were taking medications or supplements known to impact measurements of PAF and/or Lp-PLA_2_, including cholesterol lowering medications such as statins, ezetimibe, fenofibrate, niacin, orlistat, omega-3, fish oil supplements or hormone replacement therapy were excluded. Any participants who reported Asian or African ethnicity were also excluded due to these ethnic groups having lower levels of Lp-PLA_2_, possibly due to genetic polymorphisms [57–59].

With 100 participants, there was an 80% power to detect a correlation between inflammation level and food group of 0.3 or greater assuming a level of significance with less than 5% chance of type one error. Correlation of 0.3 is a medium effect size for a correlation according to Cohen [64].

### Data collection

Data, including anthropometric, biochemical, and clinical measurements, were collected at the Bond Institute of Health and Sport during a single study visit. Anthropometric data were measured in the fasted state without shoes and in light clothing. Standing height was measured using a wall mounted stadiometer, to the nearest 0.1 cm. A calibrated digital scale was used to measure weight to the nearest 0.1 kg. Waist circumference was measured six times, using a medical grade, steel, retractable tape measure with a measurement range of 10–200 cm, three times at minimum waist and three times at the umbilicus and was averaged [65]. BMI was calculated as weight in kilograms divided by height in meters squared using the formula kg/m^2^.

Sitting blood pressure was measured in the non-dominant arm, in triplicate, 2 min apart, with a clinical cuff [PC-900 Pro Vital Signs Monitor: Creative Medical]. The first blood pressure reading was disregarded and the second and third measurement were averaged [66]. Age, sex, medical history, medication and supplement intake, menopausal status, smoking status and alcohol consumption were self-reported. Levels of physical activity (PA) was assessed using the self-administered World Health Organization’s (WHO’s) Global Physical Activity Questionnaire (GPAQ) [67]. PA was assessed by the completion of 16 questions assessing time spent physically active during work, travel, and recreation in addition to sedentary time. Participant scores were then converted into metabolic equivalent (MET) minutes per weeks in accordance with the GPAQ Analysis Guide [68]. PA levels were further categorised into tertiles based on WHO’s PA recommendation using MET minutes where 0 = low, MET < 600 min/week; 1 = moderate, MET ≥ 600 to < 1500 min/week; and 2 = high, MET ≥ 1500 min/week. Methods for plasma sample collection and treatment and procedures for hsCRP, PAF and Lp-PLA_2_ assays were previously described and reported [63].

### Dietary assessment

The European Prospective Investigation into Cancer and Nutrition (EPIC) food frequency questionnaire (FFQ) [69], modified for the Australian food environment, was used to assess usual dietary intake of the participants. The EPIC FFQ was developed to measure habitual food and nutrient intake in adults and children during the past year and has been previously validated. This FFQ is a semi-quantitative paper-based questionnaire that includes two parts. The first part consists of a food list of 130 common and less common food items. The second section includes questions around breakfast cereal brand, type and quantity of milk consumed, type of fat using in cooking and baking, and the amount of visible fat on meat consumed in addition to an open section where participants can add any foods routinely consumed that was not assessed in part one. Participants responded by reporting the consumption frequency of each food item using a 9-point scale from never or less than once a month, 1–3 times per month, once per week, 2–4 times per week, 5–6 times per week, once a day, 2–3 times per day, 4–5 times per day and 6 + times per day. Each food item consumption frequency was manually entered into a spreadsheet and was converted into grams based on frequency of consumption and was further converted into serving sizes according to the Australian Guide to Healthy Eating [70]. Serving sizes for each food were then added together to form food groups. Water consumption was calculated from 3-day food diaries that were completed by participants on three consecutive days (2 weekdays and 1 weekend day) following the study visit.

The Australian Guidelines are broadly similar to other English speaking population based dietary guidance [71]. There are five principal recommendations outlined in the Australian Dietary Guidelines with guideline two recommending Australians to enjoy a wide variety of nutritious foods from five core food groups every day (which includes fruit, vegetables, grains and cereals, meat and alternatives, and milk and alternatives) and drink plenty of water [62]. Foods consumed were classified into these five food groups in accordance with the Australian Guide to Healthy Eating and water consumption was calculated in millilitres and included tea and coffee as these are considered sources of water in the dietary guidelines [62]. Some of the food groups were further subdivided into classes of foods based on known anti-inflammatory potential of the food [72–75]. Food groups and sub-groups assessed included fruit; cruciferous vegetables (including broccoli, Brussels sprouts, cabbage, cauliflower); non-cruciferous vegetables (all other vegetables excluding legumes); whole grains; refined grains; meat and poultry; fish and seafood; nuts and legumes [including nuts, peanuts, peanut butter, dried lentils, beans and peas, tofu, soya meat, textured vegetable protein (TVP) and vegetarian burgers], and dairy, both fermented (yoghurt and cheese) and non-fermented (milk). Serves of alcohol consumed, including wine, were calculated. As guideline three of the Australian Dietary Guidelines states alcohol consumption should be limited and alcohol is not one of the recommended core food groups listed in guideline two, wine was not included in the regression models as a variable. However, alcohol consumption was added to both models as a confounder.

In order to calculate energy intake for analysis, the FFQ EPIC Tool for Analysis (FETA) software was utilised. The FETA software is a cross-platform, open sourced tool that processes dietary data from the food frequency questionnaire used by the EPIC-Norfolk study [76]. The software includes ten data files containing all the individual nutrients, foods and serving sizes based on European food composition data. The original FETA files were adapted to replace the European food composition data with the Australian Food Composition Database and AUSNUT values [77, 78]. This involved manually replacing each food item’s nutrients (energy, fat, carbohydrate, protein, and sodium, potassium, and phosphorus) according to the Australian Food Composition Database.

### Data analysis

Data were analysed using SPSS version 28.0.0.0 (190) (SPSS Inc., Chicago, USA). Data were assessed for normality by examining distributions via *Q*–*Q* plots. Variables that were not normally distributed were log transformed before data analysis (PAF and hsCRP). Independent *t* tests were performed on normally distributed variables to test for differences in mean values by sex and CVD risk. Mean (SD) serves of each food group were calculated. Linear associations between food groups and markers of inflammation were assessed using Pearson’s correlation co-efficient for descriptive purposes.

Multiple linear regressions were performed to examine associations between markers of inflammation and food groups and were reported as standardized coefficients *β* and *P* values. Models included all of the food groups. Model one adjusted for age, sex, energy intake, alcohol consumption and date of data collection. Model two adjusted for variables included in model one, plus waist circumference and physical activity level. Model 2 for Lp-PLA_2_ was adjusted for LDL cholesterol due to the strong association between the LDL fraction and Lp-PLA_2_ enzyme activity [79–81]. Checks for multicollinearity were conducted using variance inflation factor (VIF) and tolerance indices. In order to adjust for multiple comparisons, the Bonferroni correction method, where the *P* value of 0.05 was divided by the number of variables being tested in the model (e.g., 0.05/8 equals a *P* value of 0.006) was used to indicate statistical significance.

To estimate the effect of a one serve change in food groups that were reported as statistically significant, the *β* coefficients were back transformed by exponentiating the coefficient. This allowed interpretation on a multiplicative scale, e.g., a back transformed value of 0.70 means a 1 serve increase in food group is associated with a 1–0.70 = 30% decrease in the inflammation measure.

## Results

### Clinical characteristics

A total of 132 people were recruited; four did not meet inclusion criteria and 28 declined to participate, leaving 100 participants who attended a study data collection visit and were included in analysis (Supplementary Fig. 1). Forty-six participants (44 classified as at high-risk for CVD, 2 classified as at low-risk) attended study visits in 2021 and 54 participants (24 classified as high-risk for CVD and 30 classified as low-risk) attended in 2022. Demographic and clinical characteristics for the total cohort, males and females, and individuals at high- versus low-CVD risk are shown in Table 1. The mean age was 49 (range 20–69) years and 92% of the cohort were Caucasian.

**Table 1:** Demographic and clinical characteristics of study subjects [63]

Characteristics	Mean ± SD or *N* (%) or median (IQR range)			*P *value^a^	Mean ± SD or *N* (%) or median (IQR range)		*P *value^a^
	Total *n* = 100	Male *n* = 31	Female *n* = 69		High Risk of CVD *n* = 68	Low Risk of CVD *n* = 32	
Age, years^b^	49 ± 13	46 ± 13	50 ± 13	0.120	53 ± 13	38 ± 14	** < 0.001**
Race, Caucasian *n* (%)	92 (92)	25 (86)	67 (94)	–	65 (96)	27 (84)	–
Male *n* (%)	31 (31)			–	21 (31)	10 (31)	–
BMI, kg/m^2b^	28.3 ± 6.5	27.41 ± 5.0	28.65 ± 7.2	0.729	30.65 ± 6.4	23.19 ± 2.7	** < 0.001**
Waist Circumference (cm) Umbilicus^b^	95.8 ± 6.7	95.99 ± 12.60	95.70 ± 18.40	0.526	102.36 ± 15.40	81.83 ± 9.15	** < 0.001**
Type 2 diabetes diagnosis %	4 (4)	3 (10)	1 (1)	–	4 (6)	0 (0)	–
Physical activity METs tertiles	1.41 ± 0.65	1.61 ± 0.72	1.32 ± 0.83	0.193	1.28 ± 0.84	1.69 ± 0.65	0.193
* n* (%) low PA	20 (20)	4 (13)	16 (23)	–	17 (25)	3 (9)	–
* n* (%) medium PA	19 (19)	4 (13)	15 (22)	–	15 (22)	4 (13)	–
* n* (%) high PA	61 (61)	23 (74)	38 (55)	–	36 (53)	25 (78)	–
PAF ng/mL^b^	7.96 (3.89–16.77)	9.95 (4.31–15.33)	6.45 (3.81–18.90)	0.814	4.84 (3.24–14.57)	13.27 (9.59–21.63)	** < 0.001**
Lp-PLA_2 _nmol/min/mL	14.91 ± 4.29	16.98 ± 4.90	13.98 ± 3.65	** < 0.001**	15.30 ± 4.42	14.09 ± 3.94	0.190
hsCRP mg/L^b,c^	0.96 (0.49–2.98)	0.93 (0.41–2.1)	1.1 (0.5–3.14)	0.392	1.79 (0.64–3.80)	0.56 (0.22–1.01)	** < 0.001**

*BMI* body mass index, *hsCRP* high-sensitivity c reactive protein, *Lp-PLA*_*2*_ lipoprotein-associated phospholipase A_2_, *mg/L* milligrams per litre, *ng/L* nanograms per litre, *nmol/min/mL* nanomoles per min per millilitre, *PA* physical activity, *PAF* platelet-activating factor, *SBP* systolic blood pressure, *SD* standard deviation

^a^Independent *T* test performed ***P* < 0.05** represents significant difference

^b^Mann Whitney *U* test performed ***P* < 0.05** represents significant difference

^c^*n* = 99

### Food group intake

Mean serves of food groups consumed are shown in Table 2. Females consumed more vegetables than males including cruciferous (1.56 ± 1.13 vs 0.84 ± 0.67, *P* < 0.001) and non-cruciferous vegetables (4.70 ± 2.24 vs. 3.61 ± 1.57, *P* = 0.017). Males consumed more refined grains than females (2.71 ± 2.35 vs. 1.50 ± 1.59, *P* = 0.003). There was no difference in consumption of any other food group between males and females.

**Table 2:** Mean consumption of serves per day of Core Food Groups according to the Australian Guide to Healthy Eating [70]

Serves/day^a^	Mean ± SD	Mean ± SD		*P* value^b^	Mean ± SD		*P* value^b^
	Total *n* = 100	Male *n* = 31	Female *n* = 69		High risk of CVD *n* = 68	Low risk of CVD *n* = 32	
Fruit	2.44 ± 1.75	2.19 ± 1.56	2.53 ± 1.82	0.357	2.24 ± 1.72	2.85 ± 1.75	0.101
Vegetables							
Cruciferous	1.33 ± 1.06	0.84 ± 0.67	1.56 ± 1.13	** < 0.001**	1.28 ± 1.12	1.44 ± .93	0.468
Non-cruciferous	4.36 ± 2.10	3.61 ± 1.57	4.70 ± 2.24	**0.017**	4.13 ± 2.02	4.86 ± 2.24	0.104
Grains and cereals							
Grains—whole	1.72 ± 1.60	2.01 ± 1.70	1.59 ± 1.55	0.231	1.70 ± 1.59	1.78 ± 1.65	0.828
Grains—refined	1.87 ± 1.93	2.71 ± 2.35	1.50 ± 1.59	**0.003**	1.81 ± 2.03	2.00 ± 1.72	0.647
Meat and alternatives							
Meat and poultry	1.75 ± 1.47	1.77 ± 0.94	1.74 ± 1.66	0.921	1.88 ± 1.01	1.48 ± 2.41	0.212
Fish and seafood	0.43 ± 0.52	0.39 ± 0.30	0.45 ± 0.60	0.594	0.45 ± 0.58	0.39 ± 0.38	0.574
Nuts and legumes	0.85 ± 0.89	0.75 ± 0.68	0.90 ± 0.98	0.441	0.69 ± 0.76	1.19 ± 1.06	**0.021**
Milk and alternatives							
Milk	1.17 ± 0.87	1.09 ± 0.75	1.21 ± 0.92	0.574	1.18 ± 0.94	1.70 ± 0.71	0.979
Yoghurt	0.23 ± 0.28	0.25 ± 0.29	0.22 ± 0.27	0.531	0.21 ± 0.29	0.26 ± 0.26	0.433
Cheese	0.25 ± 0.28	0.20 ± 0.18	0.28 ± 0.31	0.226	0.27 ± 0.31	0.21 ± 0.20	0.355

Bolded indicates significance at *P* < 0.05

^a^Serve size according to the Australian Guide to Healthy Eating [70]

^b^Independent *T* test performed

The low-risk group consumed significantly more serves of nuts and legumes than the high-risk group (1.19 ± 1.06 vs. 0.69 ± 0.76, *P* = 0.021). There was no significant difference in consumption of any other food group between high and low risk groups.

### Food groups and inflammatory markers

Pearson’s correlations between individual food groups, water, and alcohol and the inflammatory markers are shown in Table 3.

**Table 3:** Pearson’s Correlations between daily consumption of serves of core food groups, water and serves of alcohol and markers of inflammation

Food group	Log PAF		Lp-PLA_2_		Log hsCRP	
	*r*	*P* value	*r*	*P* value	*r*	*P* value
Fruits	0.030	0.770	− 0.103	0.308	− 0.139	0.169
Vegetables						
Cruciferous	− 0.211	**0.035**	− 0.206	**0.039**	− 0.108	0.289
Non-cruciferous	0.003	0.974	− 0.185	0.065	− 0.105	0.299
Total grains and cereals						
Grains—whole	− 0.052	0.610	− 0.019	0.851	− 0.143	0.159
Grains—refined	− 0.092	0.364	0.167	0.097	− 0.034	0.737
Total meat and alternative						
Red meat and poultry	− 0.091	0.365	0.025	0.808	0.127	0.211
Fish and Seafood	0.015	0.886	− 0.006	0.951	− 0.053	0.605
Nuts and Legumes	− 0.051	0.612	− 0.168	0.095	− 0.335	** < 0.001**
Total dairy						
Milk	− 0.024	0.813	0.148	0.141	0.094	0.352
Yoghurt	0.045	0.660	− 0.108	0.283	− 0.053	0.605
Cheese	− 0.235	**0.019**	− 0.259	**0.009**	− 0.012	0.903
Total water^a^	− 0.213	**0.033**	− 0.119	0.237	0.093	0.358
Coffee	0.027	0.793	0.096	0**.**343	− 0.021	0.835
Tea	− 0.230	**0.022**	− 0.080	0.426	0.031	0.761
Total alcohol	0.164	0.103	− 0.166	0.098	− 0.116	0.254
Wine	.104	0.304	− 0.252	**0.012**	− 0.070	0.494

Bolded results indicate significance at *P* < 0.05. Serving size calculated according to the Australian Guide to Healthy Eating [70]

^a^Analysis relates to millilitres consumed per day

Higher cruciferous vegetable consumption was associated with lower PAF in model 1 (*β* = − 0.22, *P* = 0.003, 95% CI [− 0.36, − 0.07]) and model 2 (*β* = − 0.27, *P* < 0.001, 95% CI [− 0.41, − 0.14]) (Table 4). Exponentiation of beta coefficients for cruciferous vegetables and PAF were 0.80 and 0.76 in model 1 and 2, respectively, thus a one serve increase in consumption of cruciferous vegetables per day was associated with 20% and 24% reduction in PAF levels.

**Table 4:** Multiple linear regression analyses of the associations between daily intake of serves of core food groups and markers of inflammation, *n* = 100

	Log PAF model 1			Log PAF model 2			Lp-PLA_2_ model 1			Lp-PLA_2_^a^ model 2			Log hsCRP^b^ model 1			Log hsCRP^b^ model 2		
	*β*	95% CI	P	*β*	95% CI	*P*	*β*	95% CI	*P*	*β*	95% CI	*P*	*β*	95% CI	*P*	*β*	95% CI	*P*
Fruit	0.08	− 0.07, 0.24	0.28	0.03	− 0.13, 0.18	0.746	− 0.04	− 0.31, 0.23	0.771	− 0.02	− 0.26, 0.23	0.890	− 0.15	− 0.42, 0.12	0.270	0.04	− 0.18, 0.26	0.722
Vegetables																		
Cruciferous	− 0.22	− 0.36, − 0.07	0.003*	− 0.27	− 0.41, − 0.14	< 0.001*	− 0.08	− 0.32, 0.17	0.525	− 0.07	− 0.30, 0.15	0.517	− 0.15	− 0.40, 0.10	0.223	0.02	− 0.18, 0.22	0.824
Non-cruciferous	0.11	− 0.05, 0.28	0.19	0.19	0.02, 0 .35	0.031	0.18	− 0.11, 0.47	0.222	0.22	− 0.05, 0.49	0.108	.19	− 0.10, 0.49	0.205	0.02	− 0.22, 0.27	0.864
Grains and cereals																		
Grains—whole	0.09	− 0.04, 0 .23	0.183	0.08	− 0.05, 0.21	0.224	− 0.10	− 0.33, 0.14	0.419	− 0.11	− 0.31, 0.10	0.319	− 0.07	− 0.31, 0.16	0.547	− 0.06	− 0.25, 0.13	0.535
Grains—refined	− 0.06	− 0.21, 0 .09	0.445	− 0.11	− 0.26, 0.04	0.149	0.14	− 0.13, 0.40	0.309	0.20	− 0.04, 0.44	0.096	− 0.01	− 0.28, 0.26	0.948	0.14	− 0.08, 0.35	0.206
Meat and Alt																		
Meat and poultry	− 0.07	− 0.18, 0 .05	0.257	− 0.10	− 0.21, 0.02	0.088	− 0.02	− 0.23, 0.18	0.872	− 0.05	− 0.24, 0.13	0.565	.04	− 0.17, 0.24	0.731	0.11	− 0.06, 0.28	0.187
Fish and Seafood	0.03	− 0.09, 0 .15	0.588	0.01	− 0.10, 0.12	0.860	0.03	− 0.17, 0.23	0.770	− 0.01	− 0.19, 0.17	0.911	− 0.09	− 0.30, 0.12	0.387	− 0.02	− 0.19, 0.14	0.778
Nuts and legumes	0.02	− 0.15, 0 .18	0.845	− 0.10	− 0.27, 0.08	0.267	− 0.20	− 0.50, 0.09	0.171	− 0.01	− 29, 0.28	0.963	− 0.51	− 0.81, − 0.22	< 0.001*	− 0.16	− 0.42, 0.09	0.203
Milk and Alt																		
Milk	0.08	− 0.04, 0 .20	0.192	0.07	− 0.05, 0.18	0.241	0.20	− 0.00, 0.41	0.058	0.12	− 0.07, 0.30	0.208	.06	− 0.15, 0.26	0.599	0.09	− 0.07, 0.26	0.270
Yoghurt	0.05	− 0.08, 0 .17	0.490	0.00	− 0.13, 0.13	0.982	− 0.10	− 0.33, 0.12	0.369	− 0.02	− 0.23, .18	.827	− 0.08	− 0.31, 0.15	0.482	0.04	− 0.14, 0.22	0.668
Cheese	− 0.14	− 0.26, − 0.01	**0.033**	− 0.15	− 0.27, − 0.03	**0.017**	− 0.26	− 0.47, 0.04	**0.024**	− 0.18	− 0.37,.02	.079	− 0.08	− 0.31, 0.14	0.457	− 0.04	− 0.22, 0.14	0.642

Serving size calculated according to the Australian Guide to Healthy Eating [70]. Model 1 adjusted for age, sex, energy intake, alcohol consumption and year of data collection. Model 2 adjusted for factors in model 1 plus waist circumference and physical activity. Bolded results indicate significance at P < 0.05

*Lp-PLA*_*2*_ lipoprotein-associated phospholipase A_2_, *PAF* platelet-activating factor

*P < 0.005 calculated using the Bonferroni correction method

^a^Model 2 adjusted for age, sex, energy intake, alcohol consumption, year of data collection, LDL cholesterol, waist circumference, and physical activity

^b^*n* = 99. Please see Additional File Table S1 for Variance Inflation Factors (VIF) and tolerance values, indicating no collinearity

Higher cheese consumption was associated with lower PAF in both models, however, these results were not significant at the *P* < 0.005 Bonferroni adjusted level (Table 4). Higher consumption of water and tea were associated with lower PAF (*r* = − 0.213 *P* = 0.033 and *r* = − 0.230 *P* = 0.022, respectively.)

Higher cheese consumption was associated with lower Lp-PLA_2_, however, this relationship was not significant at the *P* < 0.005 Bonferroni adjusted level in model 1 and was not significant after adjusting for LDL cholesterol, waist circumference and physical activity in model 2. There was a small negative correlation between wine consumption and Lp-PLA_2_ (*r* = − 0.252, *P* = 0.012).

Higher nuts and legumes consumption was associated with lower hsCRP (*β* = − 0.51, *P* < 0.001, 95% CI [− 0.81, − 0.22]) in model 1 (Table 4). However, this was no longer significant after controlling for waist circumference and physical activity in model 2. The exponentiation of beta coefficient for nuts and legumes and hsCRP was 0.60, thus a one-serve increase in consumption of nuts and legumes (e.g. 30 g nuts or 70 g dried legumes or 170 g tofu) per day was associated with 40% lower hsCRP levels.

Consumption of cruciferous vegetables was not associated with Lp-PLA_2_ or hsCRP. Consumption of cheese was not associated with hsCRP. Consumption of nuts and legumes was not associated with PAF or Lp-PLA_2_. Consumption of wine was not associated with PAF or hsCRP and tea was not associated with Lp-PLA_2_ or hsCRP.

Consumption of fruit, non-cruciferous vegetables, whole grains, refined grains, meat and alternatives, fish and seafood, milk and yoghurt and alcohol were not associated with any biomarkers of inflammation.

## Discussion

This cross-sectional study examined the relationship between core food groups and novel markers of inflammation PAF and Lp-PLA_2_, and hsCRP in 100 Australian adults at varying levels of risk of CVD. Whilst previous research has investigated other dietary patterns, notably the Mediterranean diet [45, 46, 48, 49, 82], this is the first study to focus on a dietary pattern consistent with national dietary recommendations based on food groups and these markers of inflammation. It is also the first to examine the relationship between the consumption of various healthy foods in humans using strict exclusion criteria and analysing PAF and Lp-PLA_2_ activity in a broadly Caucasian population outside of Greece [51]. A key finding from this study is that an increase in one serving (~ 75 g) of cruciferous vegetables per day was associated with 20–24% lower PAF levels. A significant inverse association was also found with cheese consumption and PAF and Lp-PLA_2_, however, these results were not significant in the fully adjusted, corrected model. Further, an increase of one serving of nuts and legumes (e.g. 30 g of nuts, 70 g dried legumes or 170 g tofu) per day was associated with 40% lower hsCRP levels. These results are promising as they highlight that simple modifications to diet may have large impacts on serum markers of inflammation.

The finding of the significant association between cruciferous vegetables such as broccoli, Brussels sprouts, cabbage, and cauliflower, and lower levels of PAF is novel and supports previous research on the role that cruciferous vegetables play in the prevention and treatment of chronic disease [83]. Numerous epidemiological studies have found higher intakes of cruciferous vegetables to be associated with a lower risk of cardiometabolic diseases [84]. Bioactive compounds in cruciferous vegetables such as glucosinolates, and their metabolites isothiocyanates, have been shown to modulate inflammation by inhibiting nuclear factor kappa B (NF-κB) and reducing cytokine secretion [85]. Further, increased PAF levels leads to increased platelet-activating factor receptor (PAFR) expression mediated via the (NF-κB) pathway [86]. There also appears to be an effect with cruciferous vegetables on platelets as a recent study found that extracts from various cruciferous vegetables were found to significantly reduce platelet activation induced by adenosine diphosphate and arachidonic acid in human platelet-rich plasma, with the highest result seen with cabbage with an 88% reduction in platelet aggregation [87].

The inverse relationship between cheese and PAF and Lp-PLA_2_ supports previous studies demonstrating that full fat dairy may not be as strongly associated with CVD risk as once thought. A recent meta-analysis examining biomarkers of dairy fat intake reported higher levels of circulating biomarkers associated with lower CVD risk [88]. However, it may be that specific types of full fat dairy play a cardioprotective role as another meta-analysis found that full fat milk was associated with an increased risk of CHD whilst cheese was inversely associated [89].

Dairy products contain polar lipids, such as phospholipids and sphingolipids, found in the milk fat globule, which have been shown to be potent inhibitors against PAF-induced platelet aggregation [90]. All sources of dairy milk contain lipids capable of inhibiting PAF-induced platelet aggregation with milk from caprine and ovine origins appearing to show the greatest anti-inflammatory effect [91]. Furthermore, a recent study has shown that consumption of bovine yoghurt enriched with olive pomace lowers biosynthetic enzymes of PAF [92]. As milk ferments to yoghurt and then to cheese, the bioactivity of the polar lipids increases the longer the fermentation process occurs, resulting in cheese having the most potent anti-inflammatory capabilities towards PAF [93].

A small association was seen with PAF and tea which aligns with research reporting that polyphenols in tea possess strong antithrombotic activities against PAF [94]. Recent research has shown there is a synergistic effect of polyphenols and polar lipids in tea which prevents oxidation and increases the anti-PAF effect [95]. A small association was seen with Lp-PLA_2_ and wine, which is in contrast to a recent study that found that wine consumption was not associated with Lp-PLA_2_ but was associated with lower PAF levels due to a reduction in biosynthetic enzymatic activity [96]. Research has shown wine has the ability to decrease postprandial platelet activity against PAF [97, 98] which may be due to polyphenols which are known for their anti-inflammatory and antithrombotic properties against PAF [99]. However, mean consumption of wine in this group was low (0.29 ± 0.60 serves per day), and mean total alcohol intake (0.55 ± 0.76 serves per day) was equivalent to ~ 0.83 to 1.10 standard drinks a day which is in line with the National Health and Medical Research Council (NHMRC) guidelines for alcohol consumption which advises adults drink no more than 10 standard drinks a week [100].

The significant association of nuts and legumes with lower levels of hsCRP aligns with recent research emphasising the positive role that plant protein plays in reducing CVD mortality [101]. Primary dietary sources of plant protein include legumes and nuts, and both appear to be associated with a reduction in CVD risk. A recent umbrella review concluded that the intake of nuts is inversely associated with the risk of CVD and a 21% reduction in risk is possible with the consumption of as little as 28 g of nuts a day [102]. However, the evidence specifically looking at nuts and inflammation is lacking; with two meta-analyses finding no association with nuts and CRP or any other inflammatory markers such as IL-6, interleukin 10, intercellular adhesion molecule 1 (ICAM-1), vascular cell adhesion molecule 1 (VCAM-1), and TNF-α [103, 104]. There is some evidence however, of nuts, in particular walnuts and pistachios, increasing Paraoxonase 1 (PON1), which is an anti-inflammatory biomarker that plays a role in the antioxidant activity of HDL and is cardioprotective [105].

Research has demonstrated that the consumption of legumes, like nuts, is inversely associated with CVD risk [106]. In studies examining legumes and inflammation, soy is often excluded or researched separately as soy has a different nutritional profile from legumes and contains a unique phytochemical called isoflavones that exhibits anti-inflammatory properties [107]. Nevertheless, both types of legumes appear to be associated with lower levels of CRP. A meta-analysis of non-soy legumes and CRP found a trend toward a significant effect on decreasing hsCRP concentrations, with the exclusion of one study (reporting on intakes of baked beans in sauce) leading to significant changes in the overall pool estimates [108]. Another meta-analysis looking solely at soy found consumption from natural soy products was associated with lower CRP, however, there was no association with products that contain soy extracts and supplements [109], which aligns with the current study’s results as only soy food intake was assessed.

The lack of association with other foods groups and PAF and Lp-PLA_2_ such as fruit and other vegetables was unexpected as previous research has found some associations with fruit and Lp-PLA_2_ [48] and Mediterranean diet type vegetables with PAF [110–112]. Despite research reporting that goat and sheep meat contain polar lipids with strong inhibitory properties against PAF-induced platelet aggregation, there was no observed link between meat consumption and PAF or Lp-PLA_2_, however, goat meat intake was not assessed directly in the FFQ [75]. No correlation was seen with fish and seafood which was surprising as fish contains polar lipids that have been shown in multiple studies to inhibit PAF-induced platelet aggregation and modulate the enzymes involved in PAF’s metabolism [113–115]. The absence of an association with PAF or Lp-PLA_2_ and nuts merits further investigation as no previous research has specifically investigated nuts and these markers, however, nut consumption increases PON1 and PON1 has been found to hydrolyse PAF [105, 116]. The lack of an association with legumes and whole grains with PAF and Lp-PLA_2_ is also interesting and warrants further research. Previous legume research has found peas to have the ability to inhibit PAF induced platelet aggregation [117]. Further, two studies reported that the substitution of whole grains and legumes for white rice significantly reduced Lp-PLA_2_ levels in people with prediabetes or type 2 diabetes, [118, 119]; however, the specific quantities consumed were not reported.

However, these results of the current study should be viewed with caution, due to potential confounding. Amongst other things, confounding from vaccinations for COVID-19 and/or infection due to the Omicron outbreak in Australia at the time of data collection, may have affected levels of PAF and Lp-PLA_2_ [63]. Briefly, levels of PAF were significantly higher in participants who had their blood sample collection in 2022, compared to 2021, which coincided with the Omicron variant COVID-19 outbreak in Australia, and a boost in vaccination rates with adenovirus vector and mRNA vaccines. Similarly, Lp-PLA_2_ levels appear to be elevated due to COVID-19 vaccination and/or infection as the there was no significant difference in levels of Lp-PLA_2_ between the high-risk and low-risk groups and the low-risk group’s data collection predominantly occurred in 2022. This phenomenon is described in more detail elsewhere [45, 63]. Alternatively, the current study findings may simply reflect that there is little or no association between other food groups and these novel biomarkers.

The lack of association between the other food groups and CRP was unexpected due to the association of healthy food groups and CRP [120]. Fruit and vegetables in particular contain numerous anti-inflammatory and anti-oxidant phytochemicals such as polyphenols and carotenoids, as well as vitamin C and E [121]. A similar study found fruit but not vegetables to be significantly associated with lower CRP [122], however, a systematic review and meta-analysis reported a significant reduction in CRP with increasing fruit and vegetable intake [123]. The lack of association between CRP and whole grains is supported by a recent systematic review of randomised controlled trials of inflammatory markers and whole grains, which found only 10 of the 32 studies examining CRP reporting significant results. In this review, nearly half the population had a pre-existing health condition which put them at risk of CVD and half were people with overweight or obesity which is similar to the current study’s population [124].

The lack of association with fish and seafood and CRP is counter to previous research which has shown that a high consumption of seafood is associated with lower rates of atherosclerotic cardiovascular disease and acute major ischemic events [125]. Healthy adults consuming at least 300 g of fish a week (3 serves) have been found to have 33% lower CRP compared to non-fish consumers [4]. In the current study, the mean intake of fish and seafood was high with the total group consuming a mean of 0.43 ± 0.52 serves per day (approximately 3 serves per week). How fish is prepared may affect the potential impact on CVD risk as a recent study found that non-fried fish was associated with lower CVD events whilst fried fish was associated with increased risk [126], however, in the current study, mean intake of fried fish (including fish cakes and fish sticks) was only 0.04 ± 0.09 serves per day.

The lack of association with milk, yoghurt and cheese and hsCRP is similar to results from a recent cross sectional study which found there was no association between fermented and non-fermented dairy intake and CRP, however, there was a significant positive association with butter [127]. A recent review of meta-analyses, systematic reviews and randomized controlled trials investigating dairy and inflammation concluded that while there is insufficient evidence to prove that dairy products are anti-inflammatory, dairy foods do not increase concentrations of biomarkers of chronic systemic inflammation [128]. A specific dairy group analysis found the intake of cheese did not have any impact on CRP levels. It may be that cheese and its bioactive components are not involved in CRP’s inflammatory pathway which is different to the pathways PAF and Lp-PLA_2_ are involved in.

Strengths of this study include the use of strict exclusion criteria to prevent confounding from medication and supplement intake, smoking and existing CVD on the novel markers of inflammation. In addition, certain ethnicities were excluded as they have been shown to have lower levels of Lp-PLA_2_ due to genetic polymorphisms which allowed for a more uniform sample for analysis. Diet was assessed using a validated FFQ and the multivariable statistical analysis using the Bonferroni adjustment was robust to minimise type 1 errors.

There were, however, some limitations. The assessment of usual diet is difficult and prone to error with FFQs often overestimating some food groups like fruit and vegetables [129]. PAF and Lp-PLA_2_ levels may have been elevated in some of the participants due to the COVID-19 vaccine and/or infection which may affect results of the relationship with food groups, however, we did adjust for this in our models [63]. Measures to control for the COVID-19 outbreak, which included isolation, quarantine, and social distancing, may have affected dietary intake. A recent global review has reported mixed results on the impact of COVID-19 lockdown on dietary intake with some studies reporting increased home baking and a reduction in intake of comfort food whilst other studies reported a reduction in fresh produce consumption and increased intake of energy dense foods [130]. Specifically in Australia, food insecurity was exacerbated [131] and young adults reported more negative and fewer positive changes in food practices during the pandemic [132] with increased energy intake especially from energy dense foods [133]. Results for PAF may have been different had we measured platelet aggregation as a measure of PAF action as seen in other research studies [44, 91, 110], rather than measuring PAF circulating blood levels using a commercially available ELISA assay. CRP has significant intra-individual variation and levels of this marker may be elevated in acute inflammation and not reflect a true relationship with food groups. The cross-sectional nature of the study prevents any causal relationships from being inferred.

In conclusion, this study found several foods to be associated with lower levels of markers of inflammation, however, different foods were associated with different markers suggesting that the bioactive components in the foods may each be involved in different inflammatory pathways. Cruciferous vegetables were significantly associated with lower PAF levels, and nuts and legumes were significantly associated with lower hsCRP levels. Cheese was inversely associated with PAF and Lp-PLA_2_ however this relationship was not significant after Bonferroni correction. Research examining food groups and the novel markers should be repeated in a non-pandemic setting in order to gain a better understanding of the true relationship between healthy food groups and PAF and Lp-PLA_2_.

### Supplementary Information

Below is the link to the electronic supplementary material.

Supplementary file1 (PDF 302 KB)

## Data Availability

Data described in the manuscript, code book, and analytic code will be made available upon request pending application and approval. Requests should be emailed to the corresponding author.
