# Supplementary material for: Associations between healthy food groups and platelet-activating factor, lipoprotein-associated phospholipase A2 and C-reactive protein: a cross-sectional study
Source: Eur J Nutr. 2023 Dec 8;63(2):445–60. doi: 10.1007/s00394-023-03277-8 (PMC10899352; doi:10.1007/s00394-023-03277-8)
Supplement: Supplementary file 1 — Supplementary file1 (PDF 302 KB) [file 394_2023_3277_MOESM1_ESM.pdf]

Associations between healthy food groups and platelet-activating factor, lipoprotein-associated phospholipase A_2 and C-reactive protein: A cross-sectional study

Carolyn J English,[1] Mark Jones,[2] Anna E. Lohning,[1] Hannah L Mayr,[1,3,4] Helen MacLaughlin,[5] Dianne P. Reidlinger[1*]

Supplementary Figure 1 Flow of participants through a study investigating the association between food groups and markers of inflammation

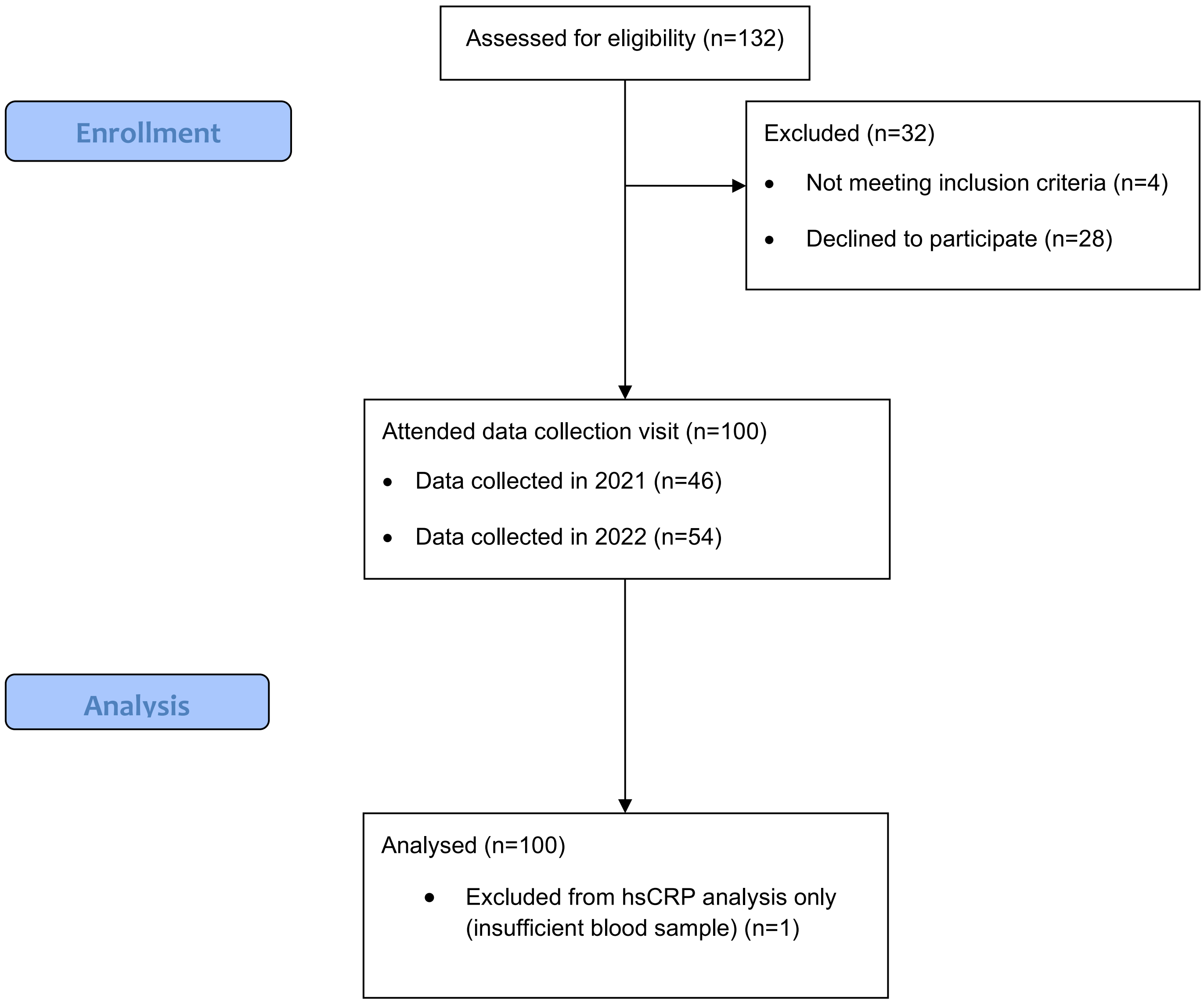

Supplementary Table 1 VIF and Tolerance of Multiple Linear Regression analysis of the associations between daily intake of serves of core food groups and markers of inflammation

	**Log PAF model 1**		**Log PAF model 2**		**$Lp\text{-}PLA_2$ model 1**		**$Lp\text{-}PLA_2$[a] model 2**		**Log hsCRP[c] model 1**		**Log hsCRP[c] model 2**	
	**VIF**	**Tolerance**	**VIF**	**Tolerance**	**VIF**	**Tolerance**	**VIF**	**Tolerance**	**VIF**	**Tolerance**	**VIF**	**Tolerance**
**Fruit**	2.09	0.48	2.23	0.45	2.09	0.48	2.22	0.45	2.06	0.48	2.20	0.45
**Vegetables**												
Cruciferous Vegetables	1.76	0.57	1.88	0.53	1.76	0.57	1.89	0.53	1.76	0.57	1.89	0.53
Non- cruciferous Vegetables	2.50	0.40	2.74	0.36	2.50	0.40	2.78	0.36	2.50	0.40	2.75	0.36
**Grains and Cereals**												
Grains - whole	1.63	0.61	1.65	0.61	1.63	0.61	1.65	0.61	1.63	0.62	1.64	0.61
Grains - refined	2.05	0.49	2.14	0.47	2.05	0.49	2.16	0.46	2.05	0.49	2.14	0.47
**Meat and Alt**												
Meat and poultry	1.22	0.82	1.65	0.79	1.22	0.82	1.28	0.78	1.22	0.82	1.27	0.79
Fish and Seafood	1.22	0.82	2.14	0.81	1.22	0.82	1.25	0.80	1.22	0.82	1.23	0.81
Nuts & legumes	2.51	0.40	1.27	0.33	2.51	0.40	3.08	0.32	2.49	0.40	2.99	0.33
**Milk & Alt**												
Milk	1.25	0.80	1.25	0.80	1.25	0.80	1.30	0.77	1.25	0.80	1.25	0.80
Yoghurt	1.48	0.68	1.56	0.64	1.48	0.68	1.60	0.63	1.47	0.68	1.55	0.64
Cheese	1.41	0.71	1.41	0.71	1.41	0.71	1.44	0.69	1.43	0.70	1.44	0.70

Model 1 adjusted for age, sex, energy intake, alcohol consumption and year of data collection. Model 2 adjusted for age, sex, energy intake, alcohol consumption, year of data collection, waist circumference, and physical activity. Bolded results indicate significance at P<.05. *P<.005 calculated using the Bonferroni correction method. Abbreviations: $Lp\text{-}PLA_2$, lipoprotein-associated phospholipase A2; PAF, platelet activating factor. [a] Model 2 adjusted for age, sex, energy intake, alcohol consumption, year of data collection, LDL cholesterol, waist circumference, and physical activity. [c]n=99
